# To Keep the Cord from Slipping in Extracting the Placenta

**Published:** 1878-06

**Authors:** 


To Keep the Cord from Slipping in Extracting the Placenta.—I have been in the habit of wrapping around my finger a small piece of rag when I extract the placenta after delivery to keep the cord from slipping. I find it to save one a great deal of time and trouble. It may not be new to all my medical brethren, but I was in consultation with a doctor of large practice of many years, who was so pleased with it that he said it ought to be generally known.—*Med. Brief.*

